# Spectrometric Analysis of the Wear from Metallic and Ceramic Dental Implants following Insertion: An In Vitro Study

**DOI:** 10.3390/ma15031200

**Published:** 2022-02-04

**Authors:** Georgios E. Romanos, Gerard A. Fischer, Zaid T. Rahman, Rafael Delgado-Ruiz

**Affiliations:** 1 Laboratory for Periodontal-Implant-Phototherapy (LA-PIP), Department of Periodontology, School of Dental Medicine, Stony Brook University, Stony Brook, NY 11794, USA; gerard.fischer@stonybrookmedicine.edu (G.A.F.); Zaid.rahman@alumni.stonybrook.edu (Z.T.R.); 2 Department of Prosthodontics and Digital Technology, School of Dental Medicine, Stony Brook University, Stony Brook, NY 11794, USA; rafael.delgado-ruiz@stonybrookmedicine.edu

**Keywords:** metals, titanium, wear, zirconia, peri-implantitis

## Abstract

Titanium wear is a growing area of interest within dental implantology. This study aimed to investigate titanium and zirconium wear from dental implants at the time of insertion using X-ray-fluorescence spectrometry (XRF) and an in vitro protocol utilizing artificial bovine bone plates. Five groups were analyzed using XRF-spectrometry: groups 1–4 (titanium implants) and group 5 (zirconia implants). The implants were inserted into two bone blocks held together by a vice. The blocks were separated, and the insertion sites were analyzed for titanium (Ti) and zirconium (Zr). Statistical descriptive analyses of Ti and Zr concentrations in the coronal, middle and apical bone interface were performed. A comparative analysis confirmed differences between the implant’s surface stability and Ti accumulation within the insertion sites of the bone block. There was a direct relationship between implant length and the quantity of titanium found on the bone block. The data generally indicates greater quantities of titanium in the coronal thirds of the implants, and less in the apical thirds. The titanium and zirconium found in the bone samples where the group 5 implants were inserted was not of statistical significance when compared to control osteotomies. The results of this study confirm wear from metallic, but not ceramic, dental implants at the time of insertion.

## 1. Introduction

Dental implants have become a popular option for the replacement of one or more missing teeth due to their versatility, survivability, biocompatibility, and predictable results [1]. Implants may be used for both single- and multiple-unit removable or fixed prosthetic dental restorations. This is an area of critical importance in dentistry due to the prevalence of edentulism in the population [2,3]. Partial and complete edentulism is known to have a negative impact on diet and nutritional intake as well as depression and self-rated health [4,5]. The surgical process of implant insertion into bone is complex. Many interactions occur within peri-implant hard and soft tissue structures, and the processes of flap and periosteum elevation at the time of insertion have a profound impact on the environment [6]. Bone density is modified by the surgical insertion of a dental implant as there is a dynamic balance between bone removal and condensation [6]. This balance exerts an important influence on the morphological features of the biomaterials used in implant therapy. Therefore, biomaterial research has become an area of great interest in implant dentistry.

Titanium is considered highly biocompatible with low toxicity, but there is growing concern about titanium particles liberated from dental implant surfaces, which become shed in the bone and peri-implant tissues [7,8]. Similarly, other materials, such as zirconium and zirconia alloys, have presented with similar surface modifications when utilized in implant dentistry [9]. A more critical review of current research reveals that there are many stages during an implant’s life, during which titanium particles may be shed from the surface into the surrounding local structures. This process may occur as early as the time of initial implant insertion, during maintenance protocols (such as ultrasonic scaling), under loading forces of mastication, and during tribo-corrosion phenomena [8,9].

The presence of titanium particles in peri-implant tissues has become an area of concern in implant dentistry. Although titanium is considered highly biocompatible, with low toxicity, immunogenicity occurs when smaller particles are leached into the body [10,11,12]. Analyses of peri-implantitis biopsies have identified titanium and dental cement as prominent foreign bodies with surrounding pro-inflammatory cells [13]. Titanium particles induce inflammation via a size-dependent macrophage-mediated immune response. Pro-inflammatory cytokines are released, such as IL-1β, IL-6, and TNF-α [14]. Pettersson et al. confirmed this proinflammatory cascade in a 2017 study. They found that Ti ions induced inflammasome activation and subsequent IL-1β release [15]. These cytokines are associated with osteoclast activation via the RANK-L/RANK/OPG pathway, which is associated with bone resorption, peri-implantitis initiation, and progression [14]. In addition to the concern over local inflammation induced by titanium particles, there is also concern regarding systemic toxicity. Studies have found that titanium particles may be ingested in saliva, after which they may enter the blood and circulatory system, deposit in tissues such as lung, liver, and splenic tissue, and cross the blood–brain barrier to enter the central nervous system [8,11,16]. It has been proposed that once titanium particles enter the cells of these tissues, they may induce DNA damage via oxidative stress [17,18,19]. Due to the potential adverse effects of titanium particles liberated from dental implant surfaces on peri-implant and systemic health, future studies should focus on better understanding their toxicity. More information is needed regarding the impact of the size of these particles as a trigger phenomenon for the further acceleration of peri-implantitis or other diseases that can result in implant failure.

Regarding zirconia implants, there are currently no available studies that evaluate the wear of zirconia dental implants after insertion. However, studies on orthopedics and prosthodontics provide some insight into the phenomena of zirconia wear [20,21,22,23]. Nogiwa et al. [20] evaluated recovered zirconia femoral heads and noted a variation in the surface roughness and clusters of phase transformation. The authors attributed these changes to the abrasive wear produced by mechanical and tribological factors. Parkes et al. [21] explained that the changes in zirconia hip replacements (increased roughness and phase transformation) were the result of unstable crystals and were not related to time. Perrichon et al. [22] assessed the alterations to zirconia femoral heads after simulated wear and aging; their results suggest that not only ageing, but friction and shocks (small, but intense, energy impacts) produced the typical wear observed in their samples. They also observed Zr debris of nanometric size released from zirconia microfractures.

The aim of this study was to investigate the process of wear from metallic (titanium) and ceramic (zirconia) implants at the time of implant insertion using XRF spectrometry and an in vitro protocol, according to which the implants were inserted into artificial bone plates. We hypothesized that traces of Ti and/or Zr would be found in the surrounding artificial bone immediately after insertion.

## 2. Materials and Methods

### 2.1. Implant Insertion into Artificial Bone

**Figure 1:**
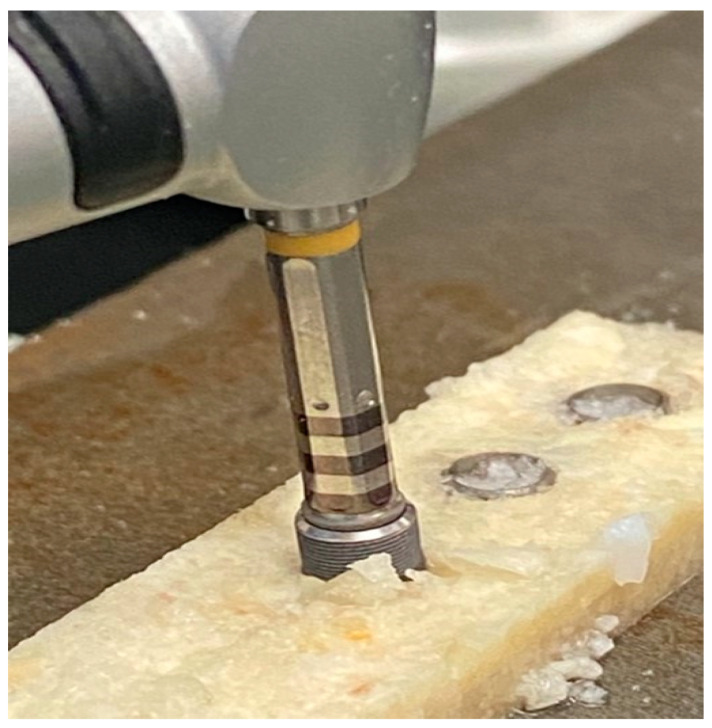
Implant insertion into junction of two artificial bovine bone plates held together by a vice.

**Figure 2:**
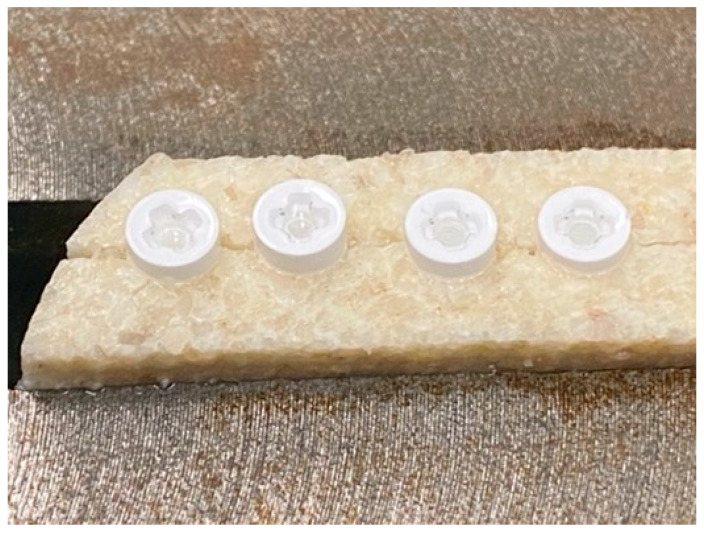
Group 5 zirconia dental implants inserted at junction of two bovine bone plates.

To analyze potential Ti and Zr accumulation in peri-implant bone in vitro, we utilized a protocol in which two separate plates of artificial bovine bone block (BoneSim^®^; Newaygo, MI, USA) were held in contact using a vice (Figure 1). The bone quality was classified according to Lekholm and Zarb as bone quality type I [24]. Implants were then inserted at the junction of the two bone plates, following the manufacturer’s guidelines on implant placement (Figure 2). Following implant insertion, the plates were removed from the vice and separated, and the areas of bone that came into contact with the implants (bone-implant-interface) during insertion were analyzed using the XRF spectrometer. The drilling process was performed in sterile distilled water (external irrigation via syringe), at an 800 RPM drilling speed. We utilized the Implantmed Plus drilling system by W&H Implex Inc^®^ (Brownstown, MI, USA). A contralateral surgical handpiece was used.

### 2.2. X-ray Fluorescence Spectrometry

Analysis of the bone samples for the presence of Ti and Zr was performed using a high-resolution energy-dispersive XRF spectrometer NEX DE (Rigaku, Austin, TX, USA), which utilizes the Windows^®^-based QuantEZ software for data processing, a 60 kV X-ray tube as the source, and a FAST SDD^®^ detector (Amptek, Bedford, MA, USA) for data collection. A semi-quantitative method of fundamental parameter (FP) analysis was completed. This analysis is considered semi-quantitative as it is a standardless method of calibration. Instead of a standard, the spectrometer software uses XRF calculations to model the matrix and calculate the concentrations of each element within a complex sample without an assayed standard [25]. The spectrometer calibration was kept up to date following the manufacturer’s guidelines. The multi-channel analyser (MCA) and library calibrations were run using the calibration sample included with the machine. The experimental set-up parameters were as follows: a helium atmosphere with a flow rate of 200.00 mL/min, DE-10 mm, a measuring time of 60 s per atomic number level (high-z, mid-z, low-z), power of 12 W, and 60 kV X-ray tube. Each bone interface was examined in three different areas (coronal, middle and apical) within a region of interest with identical focal field; therefore, six measurements were completed per implant.

It is important to note the distinction between the limit of quantification and the limit of detection of the spectrometer. When a concentration of an element is below the limit of quantification, it can be concluded that there is some level of the element that is detectable, although the chance of error is increased for the concentration calculated by the software. In other words, the limit of quantification is the lowest concentration that can be reliably measured. This is reflected by the mean values and standard deviations reported in the data.

Descriptive analysis with mean values of Ti and Zr elements (± standard deviations), were calculated and data are provided in the supplemental file.

### 2.3. Experimental Groups

**Figure 3:**
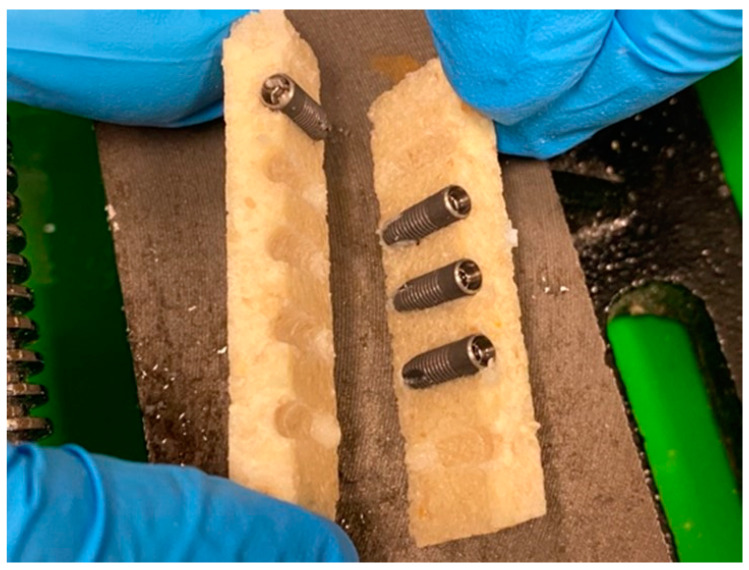
Artificial bovine bone plates after removal from the vice and separation following implant insertion, revealing areas of bone for XRF spectrometric analysis.

Titanium and zirconia implants with different designs were used for this experiment. Five experimental groups were created, as follows: group 1 (Astra OssteoSpeed^®^, Waltham, MA, USA), TiO-blasted surface, grade IV pure titanium; groups 2 (Nobel Active^®^, Yorba Linda, CA, USA) and 3 (Nobel Replace^®^, Yorba Linda, CA, USA), anodized surface, grade IV pure titanium; group 4 (Straumann Bone level tapered^®^, BLT, Basel, Switzerland), 85% Ti/15% Zr alloy, SLA-active surface, grade IV; and group 5 (Zeramex XT^®^, Miami, FL, USA), zerafil surface, hard zirconium oxide ATZ blanks. From each block formed by the two bone plates, the interfaces were examined using the spectrometer in different areas (Figure 3). Control osteotomies without implant insertion were analyzed to evaluate potential titanium levels from the drilling process (impact of the drills). This served as the control group for the experiment. In addition, analysis of the bone specimen and the water used for irrigation was performed to identify the presence of titanium and zirconium. Finally, the implant system drills were analyzed for the presence of titanium.

## 3. Results

The spectrometric analysis detected the presence of Ti on the bovine bone blocks following titanium implant insertion in dense bone. The control groups showed low levels of Ti and Zr before implant insertion. The zirconia implants group showed comparable levels of Ti and Zr detection in the osteotomies before and after the zirconia implant insertion. Although traces of other materials, such as Nickel, may have been observed in the spectrometric analysis, this study was focused on the study of titanium wear at the time of implant placement, based on previous studies [9].

### 3.1. Titanium Implants

The data showed an increase in deposited titanium for the longer implants within each implant type (e.g., group 4: 8 mm vs. 12 mm vs. 14 mm vs. 16 mm). At every third (apical, middle, coronal) of the block, titanium was deposited, although this was typically at lowest levels at the apical third.

For group 4, the coronal third showed the highest titanium levels, except for the longest (16 mm) implant, which showed a slightly higher level at the middle third (middle third 1290 ± 625 ppm vs. coronal third 1130 ± 40 ppm). Group 1 also showed a higher middle than coronal third titanium reading (2919 ± 784 ppm vs. 1951 ± 491 ppm). Both the group 2 and group 3 implants demonstrated their highest titanium deposits at the coronal third, except for group 3, with the 10 mm implant, in which the coronal third demonstrated the lowest levels (Table 1).

### 3.2. Zirconia (Zr) Implants

Spectrometric analysis of group 5, 5.5 mm × 10 mm WB ceramic implants, showed both zirconium (Zr) and titanium residues after insertion. All areas contained these two elements except for the coronal third, which lacked titanium. However, these levels were indiscernible from the control group (osteotomies without implant insertion) (Table 2).

**Figure 4:**
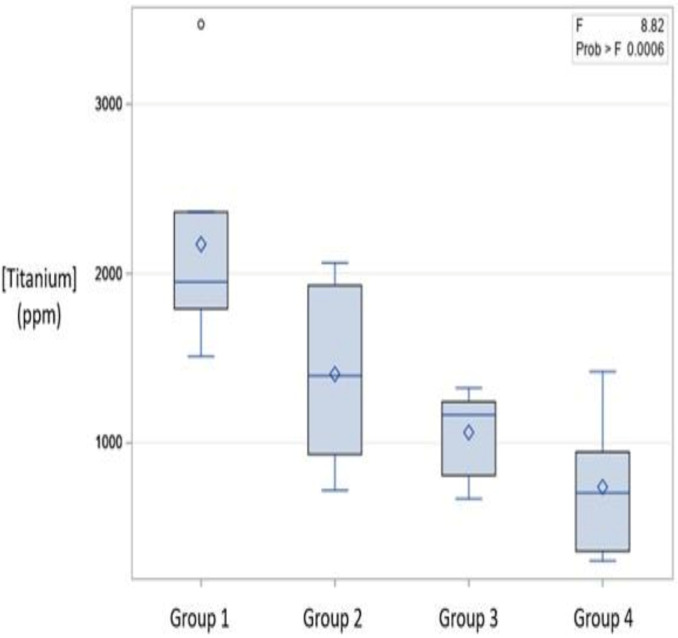
Boxplot representing minimum, maximum, mean, median, and quartile values of titanium (ppm) for each manufacturer implant. The data were taken from Table 3.

The mean values for each implant system across all lengths and areas of detection were determined (Table 3 and Figure 4). The group 1 implants (only 13 mm was tested) demonstrated the highest overall level of titanium, followed by group 2, group 3, and group 4. The group 5 implant data for titanium was not included in this comparison due to the indeterminate (non-detectable) levels of titanium deposited onto the bovine block.

### 3.3. Implant System Drills

A spectrometric analysis of the implant system drills was also performed to evaluate the potential impact of titanium particles released from the drills. The group 1 implant system drills were found to contain titanium, while titanium was not detected for the groups 2, 3 and 4 implant system drills (Table 4). These values are from the direct XRF spectrometric analysis of the implant drills rather than the control osteotomies.

## 4. Discussion

Our spectrometric semi-quantitative analysis provided information regarding the concentrations in ppm of Ti and Zr within each experimental group. However, this analysis does not provide information related to the origin and size of the particles in the sample. While many previous studies have discussed inflammation and the biological impact of titanium nanoparticles specifically, it is likely that there was a range in the size of particles detected in this study. Two key trends were observed in the data; there was a direct relationship between implant length and the concentration of titanium detected in the bone sample. Additionally, the group 1 implants, which were manufactured with a TiO-blasted surface, yielded greater concentrations of titanium in the bone samples, whereas the group 4 implants with SLA-active surfaces yielded lower titanium concentrations in the bone samples.

The results of this study support the conclusions of several studies regarding the titanium wear from implant surfaces, specifically that which occurs at the time of initial implant placement. Franchi et al. performed a study in 2004 that reported the presence of titanium particles in the peri-implant tissues just 12 weeks following initial insertion [26]. Palazzo et al. performed a histological analysis that also supported the idea that titanium particles may be liberated at the time of implant fixation, and the particles may be found up to 2.5 cm from the implant body [27]. Pettersson et al. studied the role of implant surface roughness as well as total area and diameter on the amount of titanium abraded at the time of insertion. They found that increased surface roughness created greater friction during insertion, which led to greater titanium particle detachment [28]. These results were supported by several other similar studies [26,29,30]. It was also concluded that bone implant diameter and area were less important factors in titanium particle release [28]. These conclusions, along with the results of this study, imply that masticatory forces, corrosion, and fretting against soft tissues may not be necessary for titanium wear to occur. It is possible that the friction created between the implant and bone may contribute to the observation of the early detachment of titanium particles from dental implant surfaces [9,26].

The characterization of implant surface changes can help to clarify the mechanisms and dynamics behind the elution of Ti in the body, which remains unclear at the time of writing, in order to further explain the potential mechanisms of dental implant degradation and their relation to peri-implant diseases [31]. The rationale is that leached particles can be phagocytosed by macrophages and release mediators of inflammation, which can potentially inhibit osteoblast formation, leading to bone resorption and, ultimately, the clinical loosening of implants [32,33,34].

Recent studies showed the potential risk of metallosis due to wear and tribo-corrosion contributing to the etiologic factors of peri-implant diseases. While biofilm-induced inflammation has been the most frequently discussed etiology of implant failure, this concept of metallosis, which has previously been studied in failed metal in prosthetic joints, has become an area of growing interest when studying the etiology of peri-implant disease [35]. A recent study conducted by Guo et al. [36] discussed the potential significance of soft tissue integration at the transmucosal region of dental implants. Corrosion and titanium ion leaching could have a potentially negative impact on soft tissue integration as the particles can induce inflammatory responses via the upregulation of RANK-L in the soft tissue layer, ultimately decreasing the viability of available fibroblasts [36,37,38]. Pettersson et al. compared the mucosa of periodontitis sites to the mucosa of peri-implantitis sites and found that the latter contained a greater concentration of titanium debris [39]. The mucosa of these sites was not compared to mucosa surrounding healthy implants, but the results still imply that titanium debris in the peri-implant hard and soft tissues may be a contributing factor for the progression of peri-implantitis [39].

From the prosthodontics field in a clinical study, Miura et al. [23] evaluated forty-five monolithic zirconia crowns after a period of 3.5 years. The authors found a low percentage of fractures and abrasion attributed to wear on the occlusal surface. Fuh et al. [40] studied the bone and titanium and zirconia interfaces of cylindric implants with different thread geometries. The bone stress and the interfacial sliding were evaluated. Their results showed that zirconia implants produced lower bone stresses at the cortical area compared to titanium implants and attributed their findings to the increased sliding capabilities of the zirconia implant surface. This can explain the results of the present study which showed that Zr was detected in low quantities after zirconia implant insertion compared to the detection of Ti in the titanium implant group.

Future studies should focus on preparation techniques and the alteration of implant surface manufacturing techniques to improve the stability of the biomaterial surface and decrease wear debris during implant placement procedures.

## 5. Conclusions

Within the limitations of this experiment, it can be concluded that titanium is shed from implant surfaces at the time of implant placement. This titanium wear is pronounced in the coronal third of the peri-implant bone, and less pronounced in the apical thirds of the peri-implant bone. Ceramic implants suffer low shedding from their surfaces into surrounding bone. The amount of wear is influenced by implant design, length, and material surface characteristics.

## Figures and Tables

**Table 1:** Average titanium levels collected for each titanium implant experimental group (N = 5, 30 measurements total per experimental group). Titanium levels were determined via Rigaku NEX-DE EZ-Analysis software by measuring peak intensity following X-ray spectrometry. Measurements of titanium in parts-per-million were relative to the presence of 10 other elements; Ca, P, Sr, Zn, Zr, Ni, Al, Pb, O, W. Reported level of “ND” indicated a measurement below the limit of detection for titanium by the X-ray fluorescence spectrometer.

Experimental Group	Coronal Ti Level (ppm)	Middle Ti Level (ppm)	Apical Ti Level (ppm)	Control/Osteotomy Ti Level (ppm)
Group 1–4 mm × 13 mm	1951 ± 491	2919 ± 784	1651 ± 620	304 ± 254
Group 2–4.3 mm × 10 mm	1890 ± 40	1200 ± 90	970 ± 181	123 ± 217
Group 2–4.3 mm × 11.5 mm	1675 ± 155	1086 ± 185	575 ± 103	
Group 2–4.3 mm × 15 mm	2425 ± 85	1905 ± 55	936 ± 35	
Group 3–4.3 mm × 10 mm	747 ± 52	1170 ± 130	747 ± 4	48 ± 108
Group 3–4.3 mm × 11.5 mm	1540 ± 400	1365 ± 175	882 ± 178	
Group 3–4.3 mm × 13 mm	1485 ± 145	1069 ± 81	589 ± 25	
Group 4–4.1 mm × 8 mm	494 ± 161	404 ± 404	691 ± 122	ND
Group 4–4.1 mm × 12 mm	1008 ± 104	886 ± 498	175 ± 175	
Group 4–4.1 mm × 14 mm	1095 ± 45	872 ± 709	574 ± 53	
Group 4–4.1 mm × 16 mm	1130 ± 40	1290 ± 625	264 ± 264	

**Table 2:** Average titanium and zirconium levels using group 5, 5.5 mm WB ceramic implant.

Element	Coronal Level (ppm)	Middle Level (ppm)	Apical Level (ppm)	Control/Osteotomy Level (ppm)
**Ti**	ND	136.2 ± 211	36.1 ± 112	90 ± 166
**Zr**	160.1 ± 32.9	165.3 ± 36.8	147 ± 11.1	152.4 ± 34.3

**Table 3:** Overall element detection for each implant group. The mean values for each respective group include all implant lengths tested and each area of detection (coronal, middle, apical) across all trials (N= number of implants).

Experimental Group	N	Mean (ppm)	Min (ppm)	Max (ppm)
Group 1 (Ti)	5	2174 ± 700	1511	3473
Group 2 (Ti)	5	1407 ± 534	721	2063
Group 3 (Ti)	5	1063 ± 261	670	1325
Group 4 (Ti)	5	741 ± 433	304	1422
Group 5 (Zr)	5	158 ± 21	135	216

**Table 4:** Average values of titanium concentration (ppm) of the different implant drill systems used.

Implant System	N	Average Ti Concentration (ppm)
Group 1 System	2	53,600 ± 6700
Group 2/3 System	2	Not Detected
Group 4 System	2	Not Detected

## Data Availability

The data presented in this study are available on request from the corresponding author.

## Supplementary Materials

The following supporting information can be downloaded at: https://www.mdpi.com/article/10.3390/ma15031200/s1, Supplemental File: Descriptive analysis with mean values of Ti and Zr elements (±standard deviations), were calculated and data are provided.
